# Physiological and locomotor demands during small-sided games are related to match demands and physical fitness? A study conducted on youth soccer players

**DOI:** 10.1186/s13102-022-00535-w

**Published:** 2022-07-23

**Authors:** Filipe Manuel Clemente, Ana Filipa Silva, Adam Kawczyński, Mehmet Yıldız, Yung-Sheng Chen, Sabri Birlik, Hadi Nobari, Zeki Akyildiz

**Affiliations:** 1 Escola Superior Desporto E Lazer, Instituto Politécnico de Viana Do Castelo, Rua Escola Industrial E Comercial de Nun’Álvares, 4900-347 Viana do Castelo, Portugal; 2 Research Center in Sports Performance, Recreation, Innovation and Technology (SPRINT), 4960-320 Melgaço, Portugal; 3 Instituto de Telecomunicações, Delegação da Covilhã, 1049-001 Lisbon, Portugal; 4 The Research Centre in Sports Sciences, Health Sciences and Human Development (CIDESD), 5001-801 Vila Real, Portugal; 5 Gdańsk University of Physical Education and Sport, Gdańsk, Poland; 6 Afyon Kocatepe University Sports Science Faculty, Afyonkarahisar, Turkey; 7 Department of Exercise and Health Sciences, University of Taipei, Taipei, 111 Taiwan; 8 Exercise and Health Promotion Association, New Taipei City, 241 Taiwan; 9 Tanyu Research Laboratory, Taipei, 112 Taiwan; 10 Department of Exercise Physiology, Faculty of Educational Sciences and Psychology, University of Mohaghegh Ardabili, Ardabil, 5619911367 Iran; 11 Faculty of Sport Sciences, University of Extremadura, 10003 Cáceres, Spain; 12 Department of Motor Performance, Faculty of Physical Education and Mountain Sports, Transilvania University of Braşov, 500068 Braşov, Romania; 13 Sports Science Department, Gazi University, Ankara, Turkey

**Keywords:** Football, Drill-based games, Heart rate, Global positioning systems, Athletic performance

## Abstract

**Aim:**

The purpose of the study was: (i) to analyze the relationships of physiological and locomotor demands between small-sided games (3v3 and 5v5) and official matches (11v11); (ii) to analyze the relationships between small-sided games demands and the physical fitness of youth soccer players.

**Methods:**

The observational study lasted three weeks. In the first week participants performed the 5v5 (50 × 31 and 40 × 25 m) repeatedly over four days. In the third week they repeatedly performed the 3v3 (39 × 24 and 32 × 19 m) over four consecutive days. Twenty youth soccer players (age: 16.8 ± 0.41) were tested twice for their final velocity at 30–15 Intermittent Fitness test (V_IFT_), peak speed attained at 30-m sprint test (peak speed), and anaerobic speed reserve (ASR). The heart rate responses and locomotor demands were monitored in the SSGs (3v3 and 5v5) and matches (11v11) occurring once a week. The Polar Team Pro was used as the instrument to monitor heart rate and locomotor demands. Three official matches were also monitored during the period.

**Results:**

Results revealed no significant correlations (p > 0.05) between small-sided games and match physiological or locomotor demands. However, V_IFT_ and ASR were significantly correlated with distance covered at 5v5 (r = 0.483; p = 0.031; and r = − 0.474; p = 0.035, respectively), average speed (r = 0.474; p = 0.035; and r = − 0.453; p = 0.045, respectively), while VIFT was also significantly correlated with distance covered at Z2 intensity (r = 0.510; p = 0.022).

**Conclusions:**

The results suggest that the physiological and locomotor demands occurring in small-sided games are significantly different from those occurring in official matches. Thus, physiological and locomotor similarities between small-sided games and official matches are scarce. Considering the second purpose of this study, the results suggest that VIFT and ASR are important physical fitness parameters to modulate the amount of distance covered by the players in 5v5, the average pace, and also the distance covered at high intensities.

**Supplementary Information:**

The online version contains supplementary material available at 10.1186/s13102-022-00535-w.

## Introduction

Small-sided games (SSGs) are formats of play in which coaches adjust the task constraints aiming to fit the player’s responses to specific training objectives [1]. These drill-based games turned popular in daily soccer training practice since allow to provide a physiological and locomotor stimulus while players are involved in tactical/technical challenges that simulate some of the dynamics of the formal match [2, 3]. Although natural differences between SSGs and the official match, some researchers have been pointing out the beneficial effects of these games to improve the aerobic fitness of players [4, 5]. In fact, although different from traditional running-based exercises, SSGs can be considered high-intensity interval training since (pending the formats and constraints used) can stress physiological dimensions that are responsible for improving the aerobic power [6, 7].

Although positive aspects of SSGs, there are also some threats associated with them [8]. For example, formats of play are closely related to pitch dimensions which makes that some of the smaller formats commonly used are inadequate to sustain high-intensity locomotor demands [9]. For example, high-speed running or sprinting is scarce to nonexistent in formats such as 5v5 or smaller [10, 11]. Additionally, SSGs are contextual-dependent, which means that the intra-individual variability will be always present, similarly to occurring in official matches [2].

Considering that SSGs are currently used for developing physical fitness, some questions come about their use. One of the questions is related to the relationship with physical fitness and how physical fitness may determine the player’s demands in those games. For example, it is observed in official soccer matches that the amount of distance covered, and the distance covered at high intensity are both closely related to good aerobic fitness [12, 13]. Regarding the case of SSGs, preliminary studies testing such as hypothesis revealed that aerobic capacity is related with the total amount of distance covered and high-metabolic power distance in 5v5 format [14]. Another study [15] also revealed that final velocity attained in 30–15 Intermittent Fitness Test was large to very-largely correlated with total distance, mechanical work and high-intensity running.

Besides the relevance of physical fitness to the modulation of players’ responses during SSGs, it is also important for coaches to understand if these drill-based exercises can be faced as a “similar” stimulus to the players in regard to the official match. In fact, although tactical/technical similarities between SSGs and official matches, both seem to be different in terms of physiological and locomotor demands [11, 16, 17]. For example, SSGs are played in smaller and adjusted formats which typically increases the number of accelerations and decelerations performed, while decreasing the exposure to distances covered at high intensities [10, 11]. Moreover, SSGs (the smaller ones, for example, 3v3 or 4v4) traditionally impose heart rate responses above 85%, and in some cases higher than 90% (as in extreme-sided games like 1v1 or 2v2) [18, 19]. These differences are induced by modifications to playing formats and concurrent effects with other task constraints (e.g., objective of the task, pitch configurations).

Although the differences, few studies [20] tested a simple hypothesis of analyzing how physiological and locomotor demands can be related (or not) with the same outcomes presented in official matches. This question arises from concerns about the representativity and similarity between SSGs and matches. Coaches often select SSGs to replicate the dynamics of the match and, among other, physical and physiological stimuli. However, it is important to understand if the stimulus between these exercises and matches can be really interpreted in the same way. This report may help coaches to understand which formats of play can better replicate the official match and which of them are completely different from the match. Additionally, looking for physical fitness and demands relationships can be also important, namely to understand how different formats can be more or less dependent on key physical fitness outcomes. The knowledge of that may help coaches to understand, why, how, and when to use SSGs.

Based on those reasons, the aim of the study was two-fold: (i) to analyze the relationships between physiological and locomotor demands in SSGs (3v3 and 5v5 formats) with the same measures attained in the match; (ii) to analyze the relationships between physiological and locomotor demands in SSGs and the physical fitness of youth soccer players.

## Methods

### Study design

**Fig. 1:**
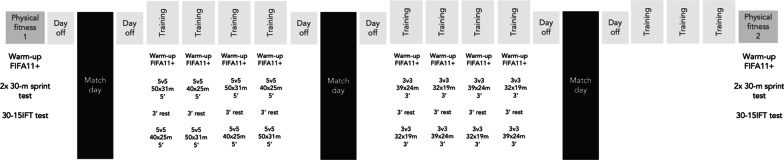
Study setting. 30-15IFT: 30–15 Intermittent fitness test

This study followed an observational study design. Players were selected by convenience sampling. The study lasted three weeks (Fig. 1) and occurred in the last third of the official season. During those weeks the players were tested twice for their physical fitness levels and monitored during eight training sessions (in which SSGs were observed) and three official matches. The study design can be observed in Table 1. The ethical committee of the University of Gazi University, Ankara, Turkey approved the protocol with code number (2021/1166, approved on 27.12.2021). The study followed the ethical standards for the research conducted on humans. All participants and their legal guardians were informed about the study and signed a free informed consent.

**Table 1:** Descriptive statistics and variability of small-sided games and match physiological and locomotor demands during the observation period

	3v3	3v3	5v5	5v5	11v11	11v11
	Mean ± SD	CV%	Mean ± SD	CV%	Mean ± SD	CV%
HRmin (bpm)	145.5 ± 17.4	12.1	134.4 ± 22.0	17.0	73.8 ± 16.8	23.5
HRave (bpm)	168.5 ± 11.9	7.1	163.1 ± 15.6	9.8	140.3 ± 20.6	15.2
HRpeak (bpm)	183.9 ± 10.4	5.7	182.8 ± 15.8	8.7	198.9 ± 16.0	8.2
Distance per minute (m/min)	132.1 ± 32.6	24.6	102.2 ± 15.9	15.8	97.5 ± 11.4	13.9
Peak speed (km/h)	20.3 ± 4.5	22.5	21.0 ± 3.0	14.4	25.3 ± 3.2	13.1
Average speed (km/h)	8.2 ± 1.9	23.3	6.4 ± 0.9	14.9	3.8 ± 1.6	50.2
Distance at Z1 (m/min)	26.0 ± 3.0	11.5	39.8 ± 6.7	16.6	35.5 ± 7.9	24.3
Distance at Z2 (m/min)	59.2 ± 25.8	43.5	27.6 ± 7.1	25.8	23.8 ± 6.1	28.3
Distance at Z3 (m/min)	21.0 ± 9.8	46.8	17.1 ± 6.9	40.3	15.5 ± 5.5	42.4
Distance at Z4 (m/min)	11.1 ± 7.5	67.3	8.9 ± 5.1	57.0	7.8 ± 3.0	46.6
Distance at Z5 (m/min)	13.1 ± 19.7	150.5	6.6 ± 8.2	123.4	4.2 ± 3.0	76.1
Dec. − 1.99 to − 1.00 m/s^2^ (n)	3.7 ± 0.5	12.3	3.7 ± 0.8	22.0	4.1 ± 1.3	33.1
Dec. − 0.99 to − 0.50 m/s^2^ (n)	5.3 ± 1.4	26.9	5.4 ± 1.1	20.4	9.0 ± 4.6	44.2
Acc. 0.50 to 0.99 m/s^2^ (n)	5.4 ± 1.5	28.6	5.5 ± 1.0	17.8	8.4 ± 4.0	42.7
Acc. 1.00 to 1.99 m/s^2^ (n)	3.6 ± 1.7	48.9	3.4 ± 1.0	31.2	3.5 ± 0.8	24.3

HR: heart rate; Dec: deceleration: ACC: acceleration

### Setting

The study occurred in the last third of the season. The first physical fitness assessment occurred on 11/02/2022 and the second on 04/03/2022 (Fig. 1).

### Participants

The a priori sample size calculation was performed using the G*power software (version 3.1.9.6). For a target of 0.7 (large correlation), a 0.05 significance, and a power of 0.8 the recommended sample size was 13 participants. We have selected by convenience a team competing in a national under-17 Turkish league. The eligibility criteria for this study were: (i) only outfield players will be enrolled; (ii) only players with no injuries, illness, or any condition that does not allow them to be part of the training sessions and physical fitness assessments during the period of observation; (iii) players cannot take any drug during the observational period; (iv) players cannot miss to any of the physical fitness assessments or training sessions in which SSGs were applied. The exclusion criteria were: (i) being a goalkeeper; (ii) missing any of the testing assessments; (iii) being injured during the observation period. Of a total of 23 soccer players bellowing to the team, only twenty were enrolled since three of them were goalkeepers.

### Physical fitness assessment

The physical fitness assessment was performed twice. On both occasions, the assessments were preceded by a 24-h rest period. The assessments occur from 17:00 to 19:00 h of the day. The environmental conditions in the first assessment were 10 °C and 67% relative humidity, while in the second assessment were 16 °C and 62% relative humidity. The assessments occurred in natural/synthetic turf. On both occasions, the last meal of the players was taken 4 h before. The assessments were preceded by a standardized warm-up protocol using the FIFA 11 + [21]. Immediately after the warm-up, the players performed two trials of 30-m linear sprint test. After performing the sprint tests, players recover 3 min and performed the 30–15 Intermittent Fitness test.

### Peak sprint speed (PSS)

The 30-m linear sprint test was applied in this study. Players were asked to start always in a split position with the same foot in front. They started with a split position, with their foot at a 30 cm distance from the starting line. The sprint started as soon as players felt ready. The players performed two trials, interspaced by 3 min of passive rest. Players have used the Polar Team pro (Polar, Finland) to determine the peak speed. The same GPS unit was kept with the same player, aiming to reduce inter-unit variability. A previous study [22] confirmed that Polar Team pro presented good levels of concurrent validity with a radar gun and also good reliability levels to estimate the peak speed while sprinting. The coefficient of variation between trials was 3.0%. The best peak sprint speed (km/h) was collected for each assessment moment (1st and 2nd) to further data treatment.

### Vinal velocity at 30–15 Intermittent Fitness test (VIFT)

The original version of 30–15 Intermittent Fitness test was applied [23]. The test presents excellent levels of reliability independently of the sex and age-group [24]. The test consists of performing 30-s runs interspaced with 15 s of rest. The test starts at 8 km/h and is progressively increasing by 0.5 km/h. Players must fit their pace with the audio record with a beep that guides them. The test ends when the player is not capable to reach the zone (3-m) related to the line which is supposed to reach. The final velocity completed by the player is registered as the main outcome of the test. The V_IFT_ was obtained in each assessment period.

### Anaerobic speed reserve (ASR)

The ASR was calculated based on the PSS subtracted by the V_IFT_ as in accordance with a previous study [25]. The ASR (km/h) in each assessment period was obtained for further data treatment.

### Small-sided games (SSGs) and matches monitoring

Since most of training protocols using SSGs are between 3v3 and 5v5 [4, 26], we have selected both for testing their relationships with match demands. In the first week of application of SSGs, the players performed the 5v5 format in four consecutive sessions, while the same occurred with the 3v3 format in the week after. Per each format, two different pitch sizes were used. One with a 100 m^2^ (1.6 length per width ratio) area per player and another with 155 m^2^ (1.6 length per width ratio) area per player. The sizes were selected based on the fact that an area closer to 90m^2^ is indicated for working counter-attack [27], while bigger areas (as 150 m^2^) are indicated to increase high intensity locomotor demands [9]. The characteristics of SSGs can be observed in Table 1. The players were organized into teams for their head coach who subjectively selected based on competitively and skill level. The players were selected for the teams in the 3v3 based on having (per team) one defender, one midfielder and one forward. In the case of 5v5, two defenders, two midfielders and one forward were selected. The players repeatedly performed the SSGs in the same team and against the same opponent’s team aiming to reduce the variability of changing opponents and teammates. No verbal encouragement was provided during the games. The formats occurred using a small goal (2 × 1 m) centered in the end line. The offside rule was not applied. Four balls were positioned around the pitches aiming to replace the ball as far as possible, every time the ball was out of boundaries. The heart rate responses and locomotor demands were monitored in all SSG using the Polar Team pro (Polar, Finland) which is confirmed for his reliability to measure the demands analyzed [22, 28]. The following measures were obtained per game: minimum heart rate (HRmin); average heart rate (HRav); peak heart rate (HRpeak); peak speed; average speed; distance covered per minute; distance covered at zone 1 (Z1: 3.00 to 6.99 km/h) per minute; distance covered at zone 2 (Z2: 7.00 to 10.99 km/h) per minute; distance covered at zone 3 (Z3: 11.00 to 14.99 km/h) per minute; distance covered at zone 4 (Z4: 15.00 to 18.99 km/h) per minute; distance covered at zone 5 (Z5: > 19.00 km/h) per minute; deceleration count (− 1.99 to − 1.00 m/s^2^) per minute; deceleration count (− 0.99 to − 0.50 m/s^2^) per minute; acceleration count (0.50 to 0.99 m/s^2^) per minute; and acceleration count (1.00 to 1.99 m/s^2^) per minute. The mean of outcomes attained for each SSG format (5v5 and 3v3) was used to further data treatment. During the observation period, the three official matches were also registered with the same instrument. The outcomes were standardized to the time in play (since they were official matches).

### Statistical procedures

Data is presented in form of mean and standard deviation. Variability of outcomes during SSGs and matches was calculated using the percentage of coefficient of variation. Visual inspection was preliminarily performed to avoid outliers. After data, data was tested for normality and homogeneity levels using the Shapiro–Wilk test and Levene's test, respectively. Correlations between physiological and locomotor demands during SSGs and matches were tested using the Pearson-product correlation test. The coefficient of correlation (r) was determined using a confidence interval of 95%. The magnitude of correlations were settled at trivial (0.0 to 0.1), small (0.1 to 0.3), moderate (0.3 to 0.5), large (0.5 to 0.7), very large (0.7 to 0.9), and nearly perfect (0.9 to 1.0). The correlations between SSGs responses and physical fitness tests were also analyzed using the Pearson-product correlation test. In this case, the 1st assessment moment (physical fitness) was correlated with the mean of performance in the 5v5 format, while the 2nd assessment moment (physical fitness) was correlated with the mean of performance in the 3v3 format. This strategy was employed to guarantee time proximity between physical fitness assessment and the game performed. The statistical procedures were executed in the SPSS software (version 28.0.0.0, IBM, Chicago, USA) for a p < 0.05.

## Results

**Fig. 2:**
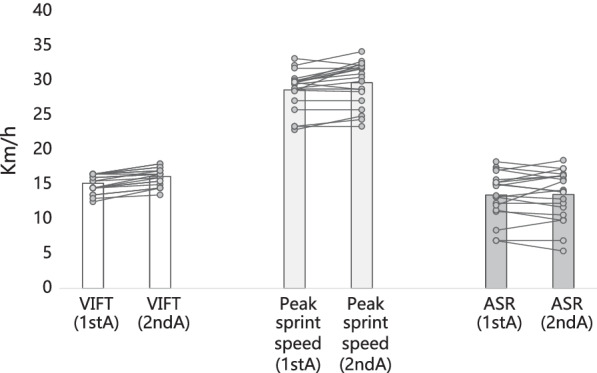
Physical fitness levels in the first (1stA) and second (2ndA) assessments. VIFT: final velocity at 30–15 Intermittent Fitness test; ASR: anaerobic speed reserve

During the period of observation, all the twenty players participated in all sessions. No missing training sessions or assessments were observed. No injuries or illnesses were also reported during the period. The twenty male soccer players (age: 16.8 ± 0.41 years; experience: 6.35 ± 0.67 years; stature: 167.85 ± 3.37 cm; body mass: 65.4 ± 6.35 kg) were assessed twice for their physical fitness levels (Fig. 2).

In the first assessment the players presented the following levels: final velocity at 30–15 Intermittent fitness test (VIFT): 15.2 ± 1.3 km/h; peak speed at 30-m sprint test: 28.7 ± 2.8 km/h; anaerobic speed reserve: 13.5 ± 3.3 km/h. In the second assessment they presented the following levels: final velocity at 30–15 Intermittent fitness test (VIFT): 16.2 ± 1.3 km/h; peak speed at 30-m sprint test: 29.7 ± 3.1 km/h; anaerobic speed reserve: 13.6 ± 3.6 km/h.

The descriptive statistics and the variability of physiological and locomotor demands during SSGs and matches can be observed in Table 1.

**Fig. 3:**
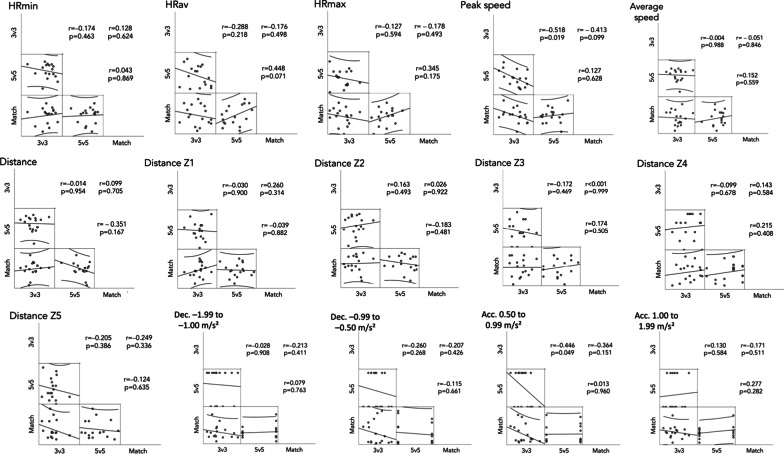
Scatter plot and correlation coefficient (r) of physiological and locomotor demands between small-sided games and match. Correlation coefficients represents the relationship between variables as in a matrix

The Fig. 3 presents the correlation coefficients between physiological and locomotor demands observed in the 3v3, 5v5 and official matches. No significant correlations were found between the SSGs and official matches (p > 0.05). However, the peak speed observed in 5v5 was largely correlated with peak speed in 3v3 (r = − 0.518 [95% confidence interval − 0.776 and − 0.085]; p = 0.019). Similarly, acceleration count (0.50 to 0.99 m/s^2^) per minute was moderately correlated between 3v3 and 5v5 (r = − 0.446 [95% confidence interval − 0.737 and 0.008]; p = 0.049).

The Table 2 presents the correlation coefficients between physical fitness and physiological and locomotor demands in 3v3 and 5v5. The results revealed that the V_IFT_ was moderately correlated with distance covered at 5v5 (r = 0.483 [95% confidence interval 0.039 and 0.757]; p = 0.031), average speed (r = 0.474 [95% confidence interval 0.028 and 0.752]; p = 0.035) and distance covered at Z2 (r = 0.510 [95% confidence interval 0.074 and 0.772]; p = 0.022). The ASR was moderately correlated with distance covered at 5v5 (r = − 0.474 [95% confidence interval − 0.752 and − 0.027]; p = 0.035), the average speed at 5v5 (r = − 0.453 [95% confidence interval − 0.740 and − 0.001]; p = 0.045) and the distance covered at Z5 (r = − 0.507 [95% confidence interval − 0.770 and − 0.070]; p = 0.022). The peak sprint speed was largely correlated with distance covered at Z5 (r = − 0.522 [95% confidence interval − 0.778 and − 0.090]; p = 0.018).

**Table 2:** Correlation coefficient between physical fitness and small-sided games physiological and locomotor demands

Format	Outcome	VIFT	PSS	ASR	Format	Outcome	VIFT	PSS	ASR
3v3	HRmin (bpm)	r = − 0.405 p = 0.076	r = − 0.195 p = 0.409	r = − 0.028 p = 0.908	5v5	HRmin (bpm)	r < 0.001 p = 0.999	r = 0.005 p = 0.984	r = 0.004 p = 0.987
3v3	HRave (bpm)	r = − 0.270 p = 0.249	r = − 0.156 p = 0.510	r = − 0.041 p = 0.862	5v5	HRave (bpm)	r = 0.229 p = 0.332	r = − 0.239 p = 0.311	r = − 0.296 p = 0.206
3v3	HRpeak (bpm)	r = − 0.068 p = 0.777	r = − 0.134 p = 0.572	r = − 0.094 p = 0.695	5v5	HRpeak (bpm)	r = 0.190 p = 0.422	r = − 0.257 p = 0.274	r = − 0.296 p = 0.205
3v3	Distance per minute (m/min)	r = 0.080 p = 0.737	r = − 0.011 p = 0.963	r = − 0.038 p = 0.874	5v5	Distance per minute (m/min)	r = 0.483 p = 0.031*	r = − 0.328 p = 0.158	r = − 0.474 p = 0.035*
3v3	Peak speed (km/h)	r = 0.005 p = 0.982	r = 0.134 p = 0.574	r = 0.115 p = 0.629	5v5	Peak speed (km/h)	r = 0.136 p = 0.567	r = − 0.049 p = 0.839	r = − 0.096 p = 0.687
3v3	Average speed (km/h)	r = 0.062 p = 0.796	r = − 0.021 p = 0.929	r = − 0.041 p = 0.865	5v5	Average speed (km/h)	r = 0.474 p = 0.035*	r = − 0.308 p = 0.187	r = − 0.453 p = 0.045*
3v3	Distance at Z1 (m/min)	r = − 0.048 p = 0.841	r = 0.024 p = 0.921	r = 0.038 p = 0.875	5v5	Distance at Z1 (m/min)	r = − 0.140 p = 0.555	r = 0.394 p = 0.086	r = 0.393 p = 0.087
3v3	Distance at Z2 (m/min)	r = 0.089 p = 0.710	r = − 0.081 p = 0.733	r = − 0.103 p = 0.667	5v5	Distance at Z2 (m/min)	r = 0.510 p = 0.022*	r = − 0.187 p = 0.430	r = − 0.364 p = 0.115
3v3	Distance at Z3 (m/min)	r = 0.055 p = 0.818	r = − 0.235 p = 0.319	r = − 0.225 p = 0.340	5v5	Distance at Z3 (m/min)	r = 0.280 p = 0.232	r = − 0.108 p = 0.650	r = − 0.205 p = 0.387
3v3	Distance at Z4 (m/min)	r = − 0.060 p = 0.800	r = − 0.152 p = 0.522	r = − 0.112 p = 0.639	5v5	Distance at Z4 (m/min)	r = 0.353 p = 0.127	r = − 0.163 p = 0.493	r = − 0.280 p = 0.231
3v3	Distance at Z5 (m/min)	r = − 0.041 p = 0.863	r = 0.285 p = 0.223	r = 0.264 p = 0.261	5v5	Distance at Z5 (m/min)	r = 0.151 p = 0.525	r = − 0.522 p = 0.018*	r = − 0.507 p = 0.022*
3v3	Dec. − 1.99 to − 1.00 m/s^2^ (n)	r = − 0.282 p = 0.229	r = − 0.067 p = 0.778	r = 0.041 p = 0.865	5v5	Dec. − 1.99 to − 1.00 m/s^2^ (n)	r = − 0.164 p = 0.489	r = 0.040 p = 0.867	r = 0.100 p = 0.675
3v3	Dec. − 0.99 to − 0.50 m/s^2^ (n)	r = − 0.251 p = 0.286	r = 0.038 p = 0.874	r = 0.122 p = 0.609	5v5	Dec. − 0.99 to − 0.50 m/s^2^ (n)	r = 0.343 p = 0.139	r = − 0.081 p = 0.735	r = − 0.206 p = 0.383
3v3	Acc. 0.50 to 0.99 m/s^2^ (n)	r = − 0.207 p = 0.382	r = 0.205 p = 0.387	r = 0.252 p = 0.283	5v5	Acc. 0.50 to 0.99 m/s^2^ (n)	r = 0.122 p = 0.608	r = − 0.057 p = 0.812	r = − 0.097 p = 0.683
3v3	Acc. 1.00 to 1.99 m/s^2^ (n)	r = − 0.086 p = 0.718	r = − 0.146 p = 0.539	r = − 0.097 p = 0.683	5v5	Acc. 1.00 to 1.99 m/s^2^ (n)	r = − 0.110 p = 0.644	r = 0.057 p = 0.812	r = 0.093 p = 0.698

HR: heart rate; Dec: deceleration: ACC: acceleration; VIFT: final velocity attained in 30− 15 Intermittent Fitness Test; PSS: peak sprint speed performed in 30-m linear sprint test; ASR: anaerobic speed reserve

**Fig. 4:**
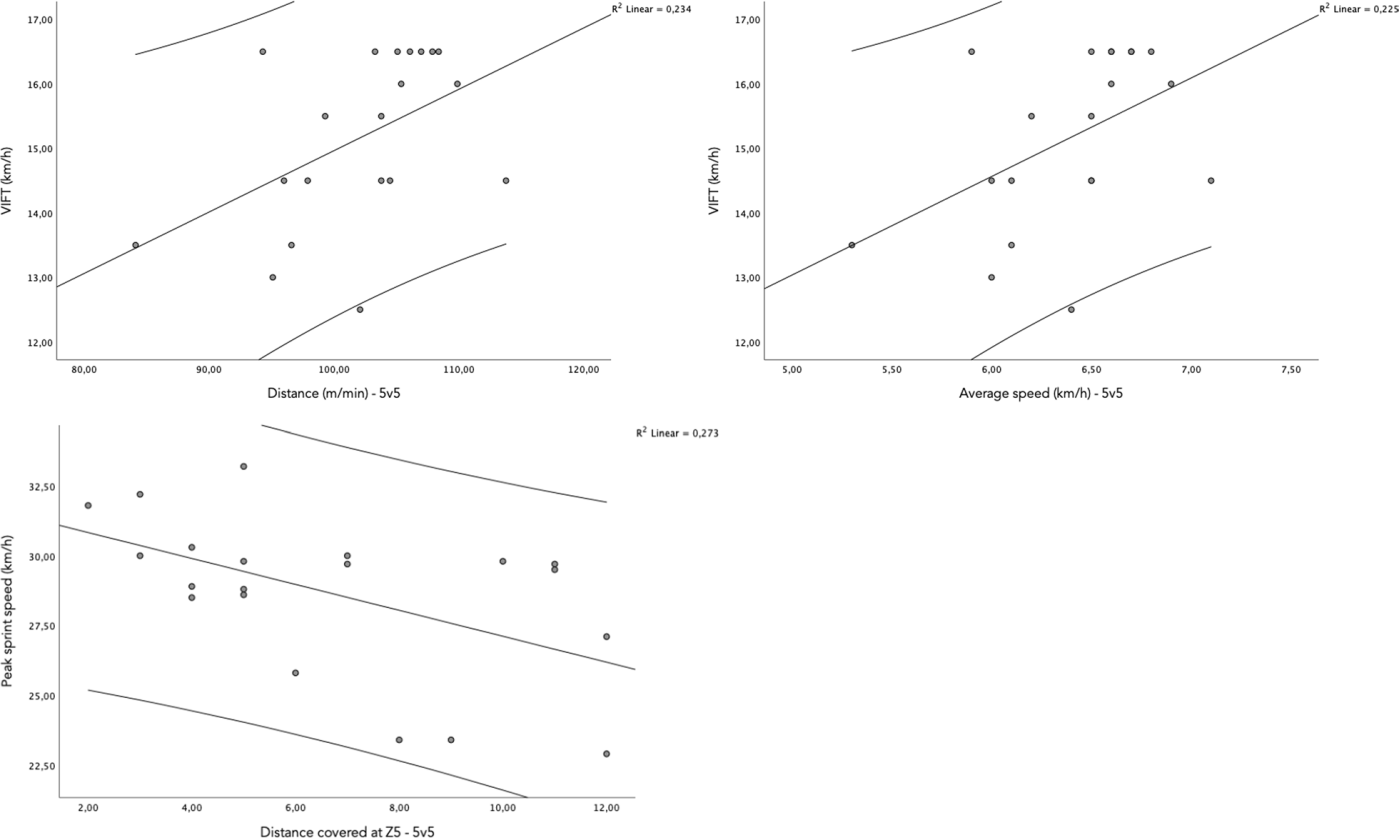
Scatter plot and coefficient of determination (R^2^) between V_IFT_ and peak sprint speed and distance, average speed and distance covered at Z5 on 5v5 format

The Fig. 4 presents the scatter plot of V_IFT_ and distance covered, and average speed attained in 5v5 format and peak sprint speed and distance covered at Z5. It is possible to observe that the coefficient of determination is R^2^ = 0.234 for distance covered and R^2^ = 0.225 for average speed. Moreover, the coefficient of determination between peak sprint speed and distance covered at Z5 was R^2^ = 0.273 (Additional file 1).

## Discussion

This study aimed to test the relationships between physiological and locomotor demands attained in SSGs and matches. Moreover, this research proposed to test the relationships between physical fitness and physiological/locomotor demands during SSGs. The main evidence of this study was that physiological and locomotor demands attained by the players during SSGs are not significantly related to the same measures occurring in official matches. Moreover, the physical fitness measures are not related to physiological and locomotor demands attained in 3v3. However, V_IFT_ and ASR were significantly correlated with distance and average speed occurring in 5v5, while ASR was also correlated with distances covered in Z5 occurring in the same format of play. Additionally, the peak sprint speed was largely correlated with distances covered in Z5.

The SSGs are typically organized in extreme (1v1), small (2v1 to 4v4), medium (5v4 to 7v7), and large (> 8v7) [29]. These differences will impact the space available to run since there is a close relationship between the format of play and the pitch dimension [30]. In fact, as longer is the field's length, the more space is available to accelerate and achieve higher speeds. This is an evidence from previous works in SSGs [31]. Since most of training protocols using SSGs are between 3v3 and 5v5 [4, 26], we have selected both for testing their relationships with match demands. Our results revealed that SSGs and match demands are not significantly correlated.

Smaller formats (1v1 to 4v4) commonly stress the heart rate responses for values around 85 and 92% of maximum heart rate [19], which is higher than the range of heart rate occurring in match which varies between 80 and 90% maximum heart rate [32]. On the other side, locomotor demands in match are typically greater with exception of acceleration frequency that often occurs in SSGs caused by the more regular change-of-direction [11, 16, 17]. These differences between SSGs and matches are expectable [33]. However, the absence of correlation represents that those attaining greater or smaller heart rate responses during the SSGs are not the same attaining in matches and vice-versa. This evidence can be justified by the fact that smaller SSGs will reduce also the position-dependency response occurring in the official match [34]. In fact, the responses of heart rate in match seems to be dependent from the playing position [35], naturally related with playing role and the locomotor demands associated with this role [36]. In smaller SSGs formats (such as 3v3 or 5v5) the structure of play will decrease since the participation and tactical behavior of the players will turn less regular and higher variable in positioning [37]. Although previous studies suggesting differences between playing positions in SSGs [38, 39], the reduction in format may conduct to a decline in natural between-playing position heterogeneity which will interfere with the demands occurring in the match [40]. Thus, the confirmed difference in heart rate and locomotor demands between playing positions occurring in official matches may be mitigated by smaller formats in which players participate similarly between them.

Another objective of the current study was to analyze the relationships between physical fitness and physiological and locomotor demands in SSGs. While none of the physical fitness measures were significantly correlated with physiological and locomotor demands in 3v3, it was possible to observe that distance covered, average pace, and distance covered at Z2 intensity were moderately and significantly correlated with VIFT, while the distance covered, and distance covered at Z5 were significantly correlated with ASR. Moreover, peak sprint speed was largely correlated with Z5. Previous studies revealed that aerobic fitness measured by the Yo-Yo Intermittent Recovery Test [14] and the 30–15 Intermittent Fitness test [15] was significantly correlated with distance covered in SSGs (namely on 5v5 and 3v3, respectively). Our research revealed that VIFT and ASR were both moderately and significantly correlated with distance covered in 5v5, while not with 3v3. In fact, as higher the VIFT as greater the distance covered, while as lower the ASR as greater the distance covered in 5v5. Normally, lower ASR is related with players with higher “endurance” profile [25] which may explain that the smaller the ASR the greater the ability to cover greater total distances. Moreover, considering the medium formats as the 5v5 increases the similarities with average distances covered per minute in official match [20], it is expectable that the aerobic fitness turns a greater participation in the ability to sustain the demands [41]. Interestingly, distance covered in Z5 (higher running intensity) was significantly and inversely correlated with ASR and peak sprint speed. Possibly, the players with greater ASR and peak speed are those with greater intermittence in actions (as in the case of wingers or forwards) on that achieve greater intensities but for small time and distance [42]. Thus, the player’s profile of not performing longer distances in high-speed running can be a playing strategy to be prepared for the moments of transitions and counter-attacks which often occurs in open-play [43].

Although the contribution of the study to the research topic, this article is not absent limitations. One of the main limitations is about the natural variability occurring in SSGs and matches which may influence the relationships with physical fitness. This can be understood as a potential source of bias. Despite that, we have tried repeated measures aiming to have more consistent evidence about the typical responses of players in those situations. A second limitation is related to the context of data collection. In fact, although the number of players involved in the experiment, all of them come from the same context. For a generalization of the findings, it is required a greater variety of participation and contexts. Thus, external validity is low and must be disclosed before any definitive conclusion. A third limitation is related to the fact that no strength and power muscle outcome was measured which should be considered in future works. Future research should extend the period of observation and increase the sample size. Moreover, report of within-players variability is a challenge for next reports.

Although the research limitations, this study is unique (as far as we know) in testing the correlations of physiological and locomotor demands between SSGs and official matches. As practical implications we can support the idea that SSGs are different from official matches and monitoring instruments should be employed to guarantee that players are exercising based on their typical needs. Moreover, it is also important to consider that as bigger the format of play, as higher the relationships with physical fitness which should be considered by the coach in the moment of distributing the players by the teams and selecting the appropriate format of play.

## Conclusions

This study revealed the 3v3 and 5v5 formats are not related to official matches while analyzing physiological and locomotor responses. However, the final velocity at 30–15 Intermittent Fitness Test and anaerobic speed reserve seems to play a dependent role with the amount of distance covered by the players in 5v5, the average pace, and also the distance covered at high intensities. On the other hand, none of the physical fitness measures tested in the current study was meaningfully related to demands in 3v3. Possibly, as bigger the SSGs turn, the higher the contribution of the locomotor profile of the player.

## Supplementary Information

**Additional file 1.** **3v3:** Dataset of GPS monitoring during the 3v3 format.

**Additional file 2.** **5v5:** Dataset of GPS monitoring during the 5v5 format.

**Additional file 3.** **Match:** Dataset of GPS monitoring during the matches.

## Data Availability

All data generated or analyzed during this study are availbe on the corresponding author.

## References

[1] Davids K, Araújo D, Correia V, Vilar L, Araú Jo D, Correia V (2013). How small-sided and conditioned games enhance acquisition of movement and decision-making skills. Exerc Sport Sci Rev.

[2] Filipe A, Clemente M, Aquino R, Praça GM, Rico M. Variability of internal and external loads and technical/tactical outcomes during small-sided soccer games: a systematic review. 2022;2021. 10.5114/biolsport.2022.107016 PMC9331334 35959343

[3] Clemente FM, Sarmento H (2020). The effects of small-sided soccer games on technical actions and skills: a systematic review. Hum Mov.

[4] Moran J, Blagrove RC, Drury B, Fernandes JFTT, Paxton K, Chaabene H (2019). Effects of small-sided games vs. conventional endurance training on endurance performance in male youth soccer players: a meta-analytical comparison. Sport Med.

[5] Hammami A, Gabbett TJ, Slimani M, Bouhlel E (2018). Does small-sided games training improve physical ftness and team-sport-specifc skills? A systematic review and meta-analysis. J Sports Med Phys Fitness.

[6] Buchheit M, Laursen PB. High-intensity interval training, solutions to the programming puzzle: Part II: anaerobic energy, neuromuscular load and practical applications. Sport Med. 2013. 10.1007/s40279-013-0066-5 23832851

[7] Buchheit M, Laursen PB (2013). High-intensity interval training, solutions to the programming puzzle: Part I: cardiopulmonary emphasis. Sports Med.

[8] Clemente FM (2020). The threats of small-sided soccer games. Strength Cond J.

[9] Castagna C, Francini L, Póvoas SCA, D’Ottavio S (2017). Long-sprint abilities in soccer: ball versus running drills. Int J Sports Physiol Perform.

[10] Clemente FM, Sarmento H, Rabbani A, Van Der Linden CMI, Kargarfard M, Costa IT (2019). Variations of external load variables between medium- and large-sided soccer games in professional players. Res Sport Med.

[11] Dalen T, Sandmæl S, Stevens TG, Hjelde GH, Kjøsnes TN, Wisløff U (2019). Differences in acceleration and high-intensity activities between small-sided games and peak periods of official matches in elite soccer players. J Strength Cond Res.

[12] Krustrup P, Mohr M, Amstrup T, Rysgaard T, Johansen J, Steensberg A (2003). The Yo-Yo intermittent recovery test: physiological response, reliability, and validity. Med Sci Sport Exerc.

[13] Aquino R, Carling C, Maia J, Vieira LHP, Wilson RS, Smith N, et al. Relationships between running demands in soccer match-play, anthropometric, and physical fitness characteristics: a systematic review. Int J Perform Anal Sport. 2020.

[14] Owen AL, Newton M, Shovlin A, Malone S (2020). The use of small-sided games as an aerobic fitness assessment supplement within elite level professional soccer. J Hum Kinet.

[15] Younesi S, Rabbani A, Clemente FM, Silva R, Sarmento H, Figueiredo AJ (2021). Relationships between aerobic performance, hemoglobin levels, and training load during small-sided games: a study in professional soccer players. Front Physiol.

[16] Casamichana D, Castellano J, Castagna C (2012). Comparing the physical demands of friendly matches and small-sided games in semiprofessional soccer players. J Strength Cond Res.

[17] Gómez-Carmona C, Gamonales J, Pino-Ortega J, Ibáñez S (2018). Comparative analysis of load profile between small-sided games and official matches in youth soccer players. Sports.

[18] Rampinini E, Impellizzeri FM, Castagna C, Abt G, Chamari K, Sassi A (2007). Factors influencing physiological responses to small-sided soccer games. J Sports Sci.

[19] Clemente FMFM, Lourenço Martins FM, Mendes RSRS, Martins FM, Mendes RSRS (2014). Developing aerobic and anaerobic fitness using small-sided soccer games: methodological proposals. Strength Cond J.

[20] Lacome M, Simpson BM, Cholley Y, Lambert P, Buchheit M (2018). Small-sided games in elite soccer: does one size fit all?. Int J Sports Physiol Perform.

[21] Bizzini M, Impellizzeri FM, Dvorak J, Bortolan L, Schena F, Modena R (2013). Physiological and performance responses to the “FIFA 11+” (part 1): is it an appropriate warm-up?. J Sports Sci.

[22] Sagiroglu İ, Akyildiz Z, Yildiz M, Clemente FM. Validity and reliability of Polar Team Pro GPS units for assessing maximum sprint speed in soccer players. 2021. 10.1177/17543371211047224.

[23] Buchheit M (2008). The 30–15 intermittent fitness test: accuracy for individualizing interval training of young intermittent sport players. J Strength Cond Res.

[24] Grgic J, Lazinica B, Pedisic Z (2020). Test-retest reliability of the 30–15 intermittent fitness test: a systematic review. J Sport Heal Sci.

[25] Sandford GN, Laursen PB, Buchheit M (2021). Anaerobic speed/power reserve and sport performance: scientific basis, current applications and future directions. Sport Med.

[26] Clemente FM, Ramirez-Campillo R, Afonso J, Sarmento H (2021). Effects of small-sided games vs. running-based high-intensity interval training on physical performance in soccer players: a meta-analytical comparison. Front Physiol.

[27] Fradua L, Zubillaga A, Caro O, Iván Fernández-García A, Ruiz-Ruiz C, Tenga A (2013). Designing small-sided games for training tactical aspects in soccer: extrapolating pitch sizes from full-size professional matches. J Sports Sci.

[28] Akyildiz Z, Yildiz M, Clemente FM. The reliability and accuracy of polar team pro GPS units;2020. 10.1177/1754337120976660.

[29] Owen AL, Wong DP, Paul D, Dellal A (2014). Physical and technical comparisons between various-sided games within professional soccer. Int J Sport Med.

[30] Hill-Haas SV, Dawson BT, Impellizzeri FM, Coutts AJ (2011). Physiology of small-sided games training in football: a systematic review. Sport Med.

[31] Clemente FM, Afonso J, Sarmento H, Manuel F, Id C, Clemente FM (2021). Small-sided games: an umbrella review of systematic reviews and meta-analyses. PLoS ONE.

[32] Dolci F, Hart NH, Kilding AE, Chivers P, Piggott B, Spiteri T (2020). Physical and energetic demand of soccer: a brief review. Strength Cond J.

[33] Clemente FM. The threats of small-sided soccer games: a discussion about their differences with the match external load demands and their variability levels. 42.

[34] Bredt SGT, Praça GM, Figueiredo LS, de Paula LLV, de Andrade AGP, Greco PJ (2016). Reliability of physical, physiological and tactical measures in small-sided soccer Games with numerical equality and numerical superiority. Braz J Kinanthropometry Hum Perform.

[35] Dellal A, da Silva CD, Hill-Haas S, del Wong P, Natali AJ, De Lima JR (2012). Heart rate monitoring in soccer: interest and limits during competitive match play and training, practical application. J Strength Cond Res.

[36] Torreño N, Munguía-Izquierdo D, Coutts A, de Villarreal ES, Asian-Clemente J, Suarez-Arrones L (2016). Relationship between external and internal loads of professional soccer players during full matches in official games using global positioning systems and heart-rate technology. Int J Sports Physiol Perform.

[37] Clemente FM, Afonso J, Castillo D, Arcos AL, Silva AF, Sarmento H (2020). The effects of small-sided soccer games on tactical behavior and collective dynamics: a systematic review. Chaos Solitons Fractals.

[38] Beenham M, Barron DJ, Fry J, Hurst HH, Figueirdo A, Atkins S (2017). A comparison of GPS workload demands in match play and small-sided games by the positional role in youth soccer. J Hum Kinet.

[39] Dellal A, Owen A, Wong DPP, Krustrup P, van Exsel M, Mallo J (2012). Technical and physical demands of small vs large sided games in relation to playing position in elite soccer. Hum Mov Sci.

[40] Clemente FM, Aquino R, Praça GM, Rico-González M, Oliveira R, Filipa Silva A (2022). Variability of internal and external loads and technical/tactical outcomes during small-sided soccer games: a systematic review. Biol Sport.

[41] Paraskevas G, Hadjicharalambous M (2018). Aerobic fitness of starter and non-starter soccer players in the champion’s league. J Hum Kinet.

[42] Orendurff MS, Walker JD, Jovanovic M, Tulchin KL, Levy M, Hoffmann DK (2010). Intensity and duration of intermittent exercise and recovery during a soccer match. J Strength Cond Res.

[43] Faude O, Koch T, Meyer T (2012). Straight sprinting is the most frequent action in goal situations in professional football. J Sports Sci.

